# Supplementary material for: Zika virus T-cell based 704/DNA vaccine promotes protection from Zika virus infection in the absence of neutralizing antibodies
Source: PLoS Negl Trop Dis. 2024 Oct 17;18(10):e0012601. doi: 10.1371/journal.pntd.0012601 (PMC11521268; doi:10.1371/journal.pntd.0012601)
Supplement: S1 Table — Amino acid sequences in red represent amino acids that differ from the antigenic sequence of the immunizing poly-epitope. Underlined sequences represent amino acid sequences having at least 80% sequence identity with the immunizing ZIKV-NS or DENV1-NS poly-epitopes. Numbers in brackets indicate the percentages of identity with the antigenic peptides from the immunizing poly-epitopes. (DOCX) [file pntd.0012601.s001.docx]

**Supplementary Table 1: ZIKV-NS and DENV1-NS T cell epitopes:**

**HLA-A*2402 transgenic mice immunized with ZIKV-NS poly-epitope**

- ZV 1634: AGTSGSPILDKCGRV

- DENV1: PGTSGSPIVNREGKI (53.3%)

- DENV2: PGTSGSPIIDKKGKV (73.3%)

- ZV 1790: HFTDPSSIAARGYIS

- DENV1: HFTDPASIAARGYIS (93.3%)

- DENV2: HFTDPASIAARGYIS (93.3%)

- ZV 1794: PSSIAARGYISTRVE

- DENV1: PASIAARGYISTRVG (86.6%)

- DENV2: PASIAARGYISTRVE (93.3%)

- ZV 1798: AARGYISTRVEMGEA

- DENV1: AARGYISTRVGMGEA (93.3%)

- DENV2: AARGYISTRVEMGEA (100%)

- ZV 1822: PGTRDAFPDSNSPI

- DENV1: PGSVEAFPQSNAVI (53.3%)

- DENV2: PGSRDPFPQSNAPI (73.3%)

- ZV 1826: DAFPDSNSPIMDTEV

- DENV1: EAFPQSNAVIQDEER (53.3%)

- DENV2: DPFPQSNAPIIDEER (60%)

- ZV 2352: GMPFYAWDFGVPLLM

- DENV1: GWPISKMDIGVPLLA (53.3%)

- DENV2: GWPLSKMDIGVPLLA (53.3%)

- ZV 2356: YAWDFGVPLLMIGCY

- DENV1: SKMDIGVPLLALGCY (60%)

- DENV2: SKMDIGVPLLAIGCY (66.6%)

- ZV 2360: FGVPLLMIGCYSQLT

- DENV1: IGVPLLALGCYSQVN (66.6%)

- DENV2: IGVPLLAIGCYSQVN (73.3%)

- ZV 2384: LLVAHYMYLIPGLQA

- DENV1: MLVAHYAIIGPGLQA (66.6%)

- DENV2: LLVAHYAIIGPGLQA (73.3%)

- ZV 2388: HYMYLIPGLQAAAAR

- DENV1: HYAIIGPGLQAKATR (60%)

- DENV2: HYAIIGPGLQAKATR (60%)

- ZV 2392: LIPGLQAAAARAAQK

- DENV1: IGPGLQAKATREAQK (66.6%)

- DENV2: IGPGLQAKATREAQK (66.6%)

- ZV 2805: IRSEHAETWFFDENH

- DENV1: IKNEHKSTWHUDEDN (46.6%)

- DENV2: IKQEHETSWHYDQDH (40%)

- ZV 2809: HAETWFFDENHPYRT

- DENV1: HKSTWHUDEDNPYKT (53.3%)

- DENV2: HETSWHYDQDHPYKT (46.6%)

- ZV 2829: SYEAPTQGSASSLIN

- DENV1: SYEVKPSGSASSMVN (60%)

- DENV2: SYETKQTGSASSMVN (60%)

- ZV 2833: PTQGSASSLINGVVR

- DENV1: KPSGSASSMVNGVVR (66.6%)

- DENV2: KQTGSASSMVNGVVR (66.6%)

**HLA-A*2402 transgenic mice immunized with DENV1-NS poly-epitope**

- DV 1757: IIMDEAHFTDPSSIA

- DENV2: IIMDEAHFTDPSSIA (100%)

- ZIKV: YIMDEAHFTDPSSIA (93.3%)

- DV 2032: QYSDRRWCF

- DENV2: NYADRRWCF (77.7%)

- ZIKV: TYTDRRWCF (77.7%)

- DV 2765: VLRIIGQRIENIKHE

- DENV2: NLDIIGKRIEKIKQE (73.3%)

- ZIKV: NMKIIGNRIERIRSE (53.3%)

- DV 2769: IGQRIENIKNEHKST

- DENV2: IGKRIEKIKQEHETS (60%)

- ZIKV: IGNRIERIRSEHAET (60%)

- DV 2793: KTWAYHGSYEVKPSG

- DENV2: KTWAYHGSYETKQTG (80%)

- ZIKV: RTWAYHGSYEAPTQG (66.6%)

**HLA-B*0702 transgenic mice immunized with DENV1-NS poly-epitope**

- DV 1678: RYLPAIVERAI

- DENV2: RYLPAIVREAI (100%)

- ZIKV: RVLPEIVREAI (81.8%)

- DV 1785: AAAIFMTATPPGSVE

- DENV2: AAGIFMTATPPGSRD (80%)

- ZIKV: AAAIFMTATPPGTRD (80%)

- DV 2319: ILMGLDKGWPISKMD

- DENV2: VLMGLGKGWPLSKMD (80%)

- ZIKV: VLFGMGKGMPFYAWD (40%)

- DV 2765: VLDIIGQRIENIKHE

- DENV2: NLDIIGKRIEKIKQE (73.3%)

- ZIKV: NMKIIGNRIERIRSE (60%)

- DV 2769: IGQRIENIKNEHKST

- DENV2: IGKRIEKIKQEHETS (60%)

- ZIKV: IGNRIERIRSEHAET (60%)

- DV 2793: KTWAYHGSYEVKPSG

- DENV2: KTWAYHGSYETKQTG (80%)

- ZIKV: RTWAYHGSYEAPTQG (66.6%)

- DV 2797: YHGSYEVKPSGSASS

- DENV2: YHGSYETKQTGSASS (80%)

- ZIKV: YHGSYEAPTQGSASS (73.3)

- DV 2882: KPRICTREEF

- DENV2: TPRMCTREEF (80%)

- ZIKV: KPRVCKTEEF (80%)

**HLA-B*0702 transgenic mice immunized with ZIKV-NS poly-epitope**

- ZV 20: GVARVSPFGGLKRLP

- DENV1: ARNRVSTVSQLAKRF (26.6%)

- DENV2: ERNRVSTVQQLTKRF (26.6%)

- ZV 1702: KTRRVLPEIVREAIK

- DENV1: KTRRYLPAIVREAIK (86.6%)

- DENV2: KTKRYLPAIVREAIK (86.6%)

- ZV 1722: VILAPTRVVAAEMEE

- DENV1: LVLAPTRVVASEMAE (73.3%)

- DENV2: LILAPTRVVAAEMEE (93.3%)

- ZV 2829: SYEAPTQGSASSLIN

- DENV1: SYEVKPSGSASSMVN (60%)

- DENV2: SYETKQTGSASSMVN (60%)

- ZV 2905: ELGKHKRPRVCTKEE

- DENV1: FLSRNKKPRICTREE (53.3)

- DENV2: ELGKKKTPRMCTREE (73.3%)

**HLA-B*0702 and -B*0702/IFNAR mice immunized with DENV1-NS poly-epitope**

- DV 1785: AAAIFMTATPPGSVE

- DV 2319: ILMGLDKGWPISKMD

- DV 2765: VLDIIGQRIENIKHE

- DV 2769: IGQRIENIKNEHKST

- DV 2793: KTWAYHGSYEVKPSG

- DV 2797: YHGSYEVKPSGSASS

- DV 2882: KPRICTREEF

- DV 1678: RYLPAIVREAI

**HLA-B*0702 and -B*0702/IFNAR mice immunized with ZIKV-NS poly-epitope**

- ZV 20: GVARVSPFGGLKRLP

- ZV 24: VSPFGGLKRLPAGLL

- ZV 1702: KTRRVLPEIVREAIK

- ZV 1706: VLPEIVREAIKTRLR

- ZV 1722: VILAPTRVVAAEMEE

- ZV 2829: SYEAPTQGSASSLIN

- ZV 2905: ELGKHKRPRVCTKEE

- ZV 2909: HKRPRVCTKEEFINK
