# Supplementary figures and images for: Zika virus T-cell based 704/DNA vaccine promotes protection from Zika virus infection in the absence of neutralizing antibodies
Source: PLoS Negl Trop Dis. 2024 Oct 17;18(10):e0012601. doi: 10.1371/journal.pntd.0012601 (PMC11521268; doi:10.1371/journal.pntd.0012601)

## Gating Strategy: Lymphocytes, single cell, CD3+ CD8+

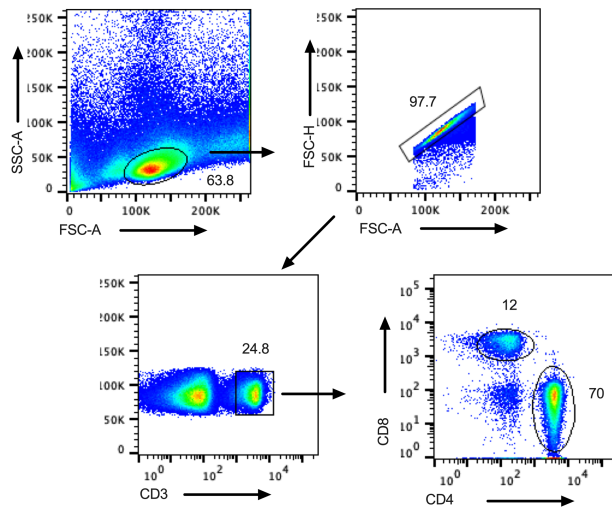

## HLA-A\*2402 day 10

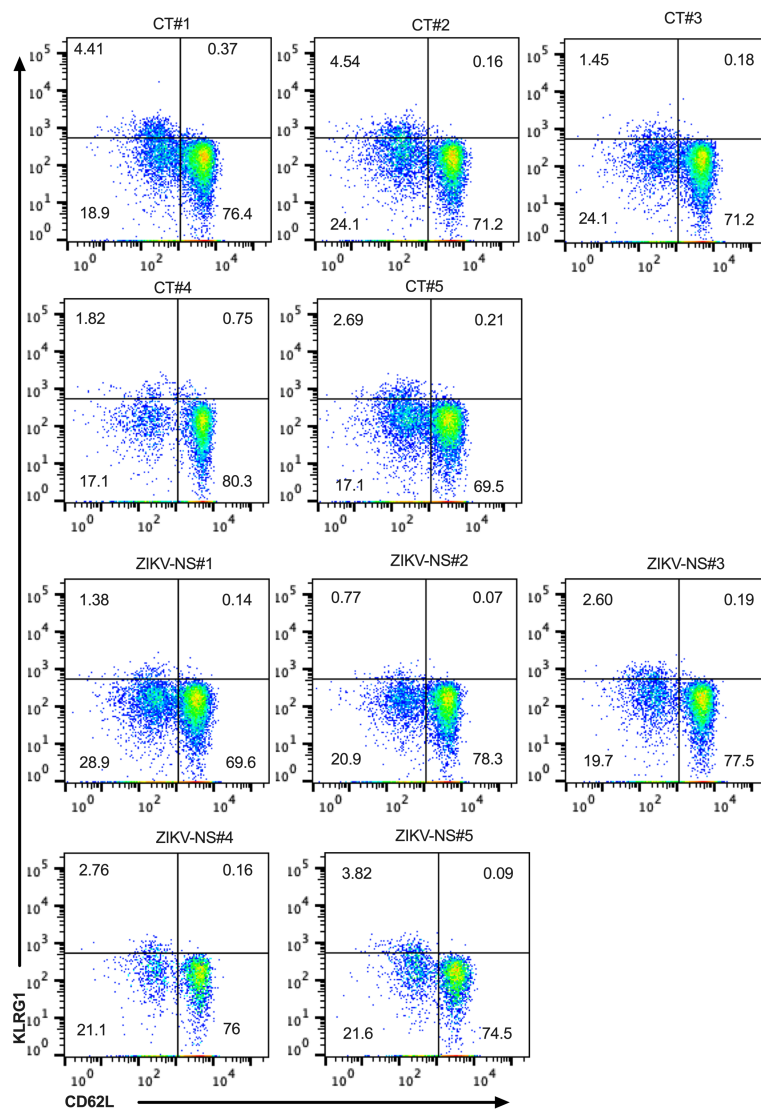

# HLA-A\*2402 day 24

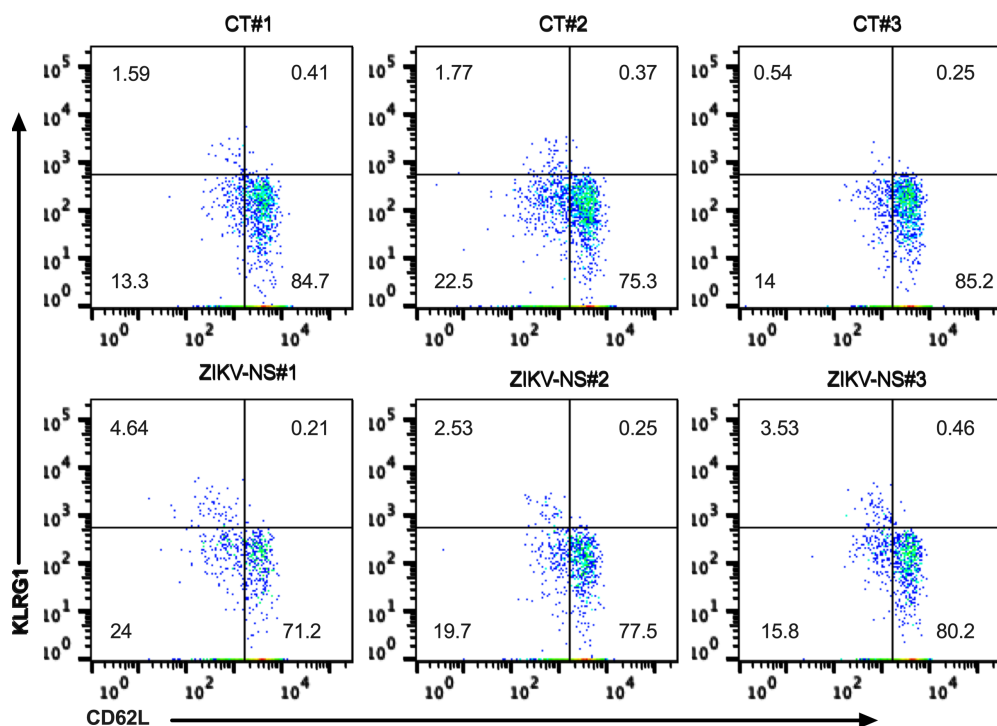

# HLA-A\*0201 day 10

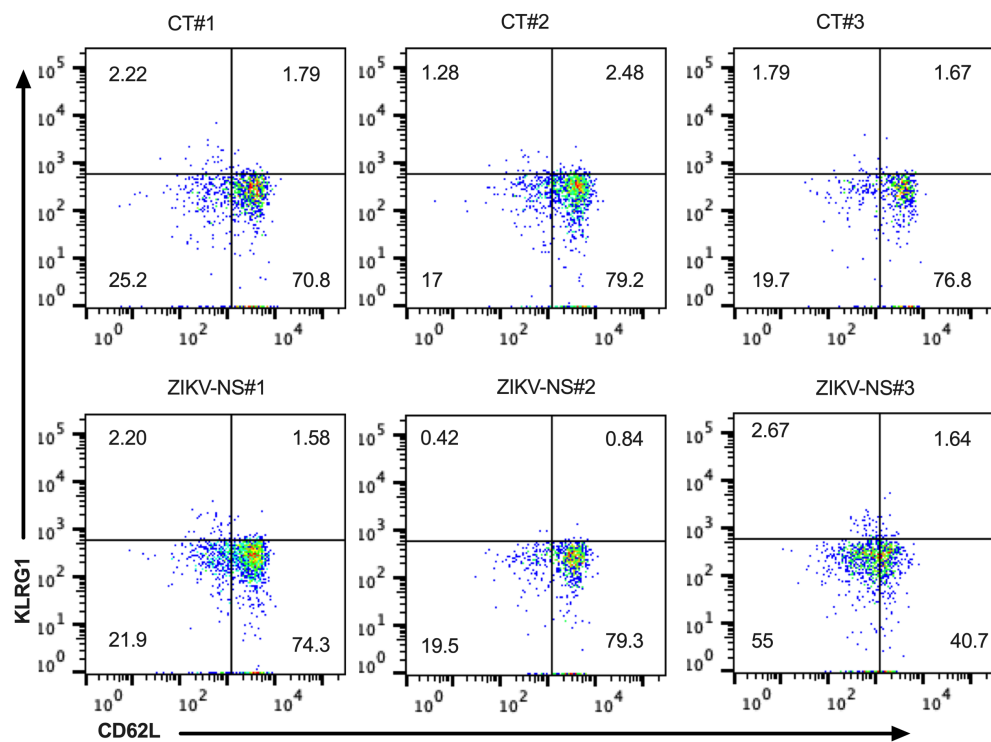

HLA-A\*0201 day 24

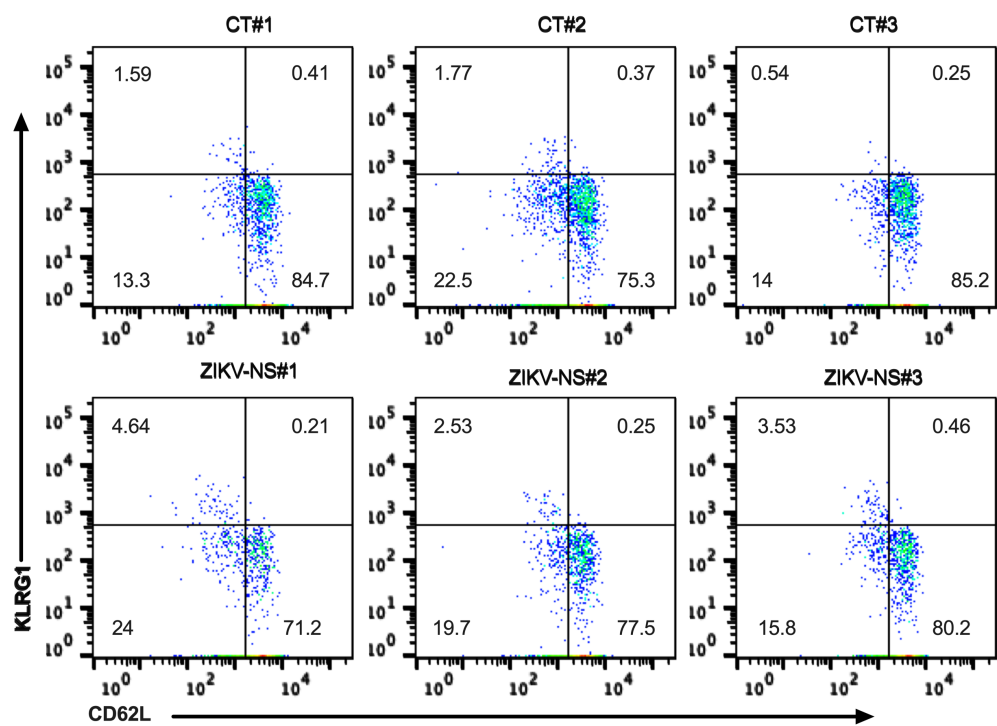

Supplement: S1 Fig — HLA-A*2402 mice immunized with ZIKV-NS or control vector, were tested at day 10 after the secondary immunization (5 mice per group), and at day 24 after the secondary immunization (3 mice per group). HLA-A*0201 mice were immunized with ZIKV-NS or control vector and tested at day 10 and day 24 after the secondary immunization (3 mice per group). (PDF) [file pntd.0012601.s002.pdf]

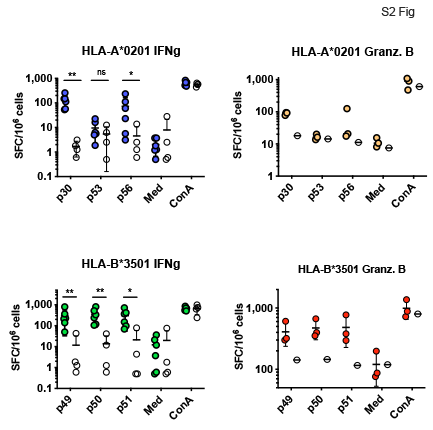

Supplement: S2 Fig — Closed and open symbols represent the animals immunized with 704 formulated DENV1-NS and the control plasmid, respectively. All the animals were immunized by intramuscular injection (2x50μg) at day 0 and day 21, and spleen cells were tested for IFN-γ or Granzyme B secretion by ELISpot assay 10 days after the second injection. Individual mice were tested in parallel with different peptides at 2μg/ml and with concanavalin A at 5μg/ml, final concentration. Peptides p30, p53, p56, and p49, p50, p51 have been characterized previously (ref 49). Lines represent mean and SEM. (n varied between 4 and 6 mice/group except for HLA-B*3501 transgenic mice with n = 3 and 1 for DENV1-NS and control plasmid, respectively. Differences between mice immunized with DENV1-NS and the control plasmid were evaluated using non-parametric Mann-Whitney U-test (*p<0.05, **p<0.01). (TIF) [file pntd.0012601.s003.tif]

S3 Fig

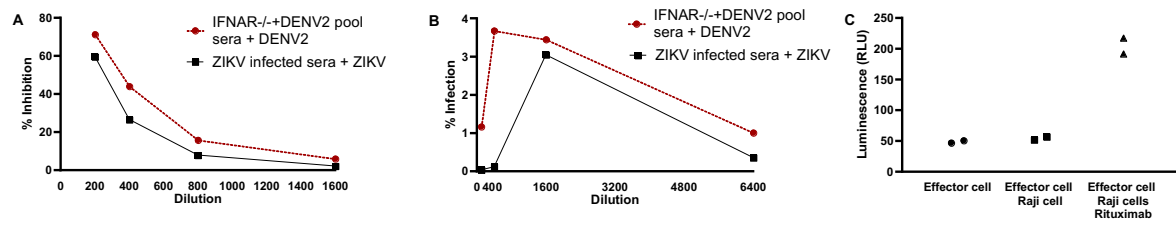

Supplement: S3 Fig — (A) flow cytometry-based neutralization assay measuring the percentage of infection. Vero CCL were in vitro infected with DENV-2 (red dot line) and ZIKV (black line) in presence or absence of serially diluted IFNAR-/- DENV2 challenged and ZIKV infected sera (B) Antibody-dependent enhancement assay. U937 cells were in vitro infected with DENV-2 (red dot line) and ZIKV (black line) in presence or absence of serially diluted IFNAR-/- DENV2 challenged and ZIKV infected sera. (C) Antibody-dependent cellular cytotoxicity assay. Jurkat reporter cells (effector cells) were incubated with Raji cell (target cells) in presence or absence of rituximab (anti-CD20) mAb. The data is shown as luminescence (RLU). (PDF) [file pntd.0012601.s004.pdf]
