# Supplementary material for: Zika virus T-cell based 704/DNA vaccine promotes protection from Zika virus infection in the absence of neutralizing antibodies
Source: PLoS Negl Trop Dis. 2024 Oct 17;18(10):e0012601. doi: 10.1371/journal.pntd.0012601 (PMC11521268; doi:10.1371/journal.pntd.0012601)
Supplement: Attachment — Submitted filename: Responses to the reviewers.docx [file pntd.0012601.s005.docx]

**Plos Negleted Tropical Diseases**	Paris July 11, 2024

Dear Editor,

Thank you for the opportunity to revise and to resubmit our manuscript "Zika virus T-cell based 704/DNA vaccine promotes protection from Zika virus infection in the absence of neutralizing antibodies" (PNTD-D-24-00451). We have modified the manuscript in response to the comments from the reviewers and believe our modifications have strengthened the results, presentation, and interpretation of our study, as recommended. Below we have detailed how we addressed the reviewer’s concerns.

**Responses to the reviewers:**

**Reviewer #1:**

1. The concept of original antigenic sin has been mentioned in the introduction, page 5 line 2-6. We have also added a reference (35) for the potential role of cross-reactive T cells in dengue disease severity following secondary DENV infection.

2. We agree with the reviewer: the sentence page 4 line 18-19 “In more general terms, ZIKV-specific CD8 T cells are an important correlate a protection for ZIKV infection” could be confusing. We have replaced this sentence line 21-22 by “Together these observations argue in favor of a protecting role of ZIKV-specific CD8 T cells against ZIKV infection”.

Concerning the beneficial role of CD4 T cells, we have added a paragraph, page 4 line 22-24 and line 26-32, including the 2 references (32, 33) suggested by the reviewer, describing the role of CD4 T cells in controlling DENV and ZIKV viremia and the quality of the antibody response.

3. Concerning the immunization with the empty vector control, in all the experiments, both to quantify the immunogenicity of the ZIKV-NS and the DENV1-NS constructs (Fig. 1 and Fig. 3), and to evaluate the immune protection (Fig. 5) we have always added in our experimental protocol a control group consisting in the injection of 704 formulated with empty pVAX1.

The ELISpot results obtained with the empty vector has been included in the Fig. 1 and Fig. 3 (page 25 line 8-11).

4. For the cross-protection between ZIKV and DENV1 induced after vaccination with the 2 different constructs, the spleen cells of animals immunized with the ZIKV-NS (or DENV1-NS) construct have been tested only against the overlapping peptides that cover the sequence of the immunizing construct.

We agree that testing the splenocytes against peptides of the non-immunizing flavivirus (i.e. immunization with ZIKV-NS and testing with DENV peptides or vice versa) and/or testing with invariant peptides across the dengue serotypes would be informative, however, this would require a larger number of mice included in the study. In addition, we agree that it would have been also informative to test the immune protection against ZIKV infection in mice immunized with DENV1-NS. However, we were unable to carry out these experiments, mainly due to the limiting number of HLA-B*0702 transgenic mice available. These proposed experiments will be the subject of future follow up studies. We have mentioned this limitation in the discussion (page 18, line 33-37 and page 19, line 1-3).

5. We agree with the reviewer’s suggestions, and we have analyzed the phenotype of CD8 T cells, induced after immunization with 704 formulated with ZIKV-NS or the empty construct pVAX1.

The CD62L and KLRG1 markers were used to identify effector memory CD8 T cells activated after immunization of HLA-A*0201 and HLA-A*2402 transgenic mice with the ZIKV-NS. Knowing that effector memory CD8 T cells have been identified as KLRG1hi CD62Llo (Ref.59), the expression of these markers was measured at day 10 and day 24 after the second immunization with ZIKV-NS. As shown in Fig. 4 and Supplementary Fig. S1, flow cytometry analyses on gated CD3+ CD8+ T cells revealed a slight and a significant increase in the percentage of KLRG1hi CD62Llo effector memory CD8 cells in HLA-A*2402 and HLA-A*0201 mice, respectively, at day 24 after the secondary immunization. We have included these data in the revised version of the paper (Fig 4 and S1 Fig., page 14 line 8-25).

We have also measured by ELISpot assay the ability of T cells to degranulate and produce the enzyme Granzyme B. These data, performed on HLA-A*0201 and -B*3501 transgenic mice, clearly show that T cells can degranulate and produce Granzyme B, in response to antigenic peptides (Supplementary Fig. S2).

**Reviewer #2:**

In this paper, as the challenge experiments with DENV and ZIKV were performed in a BSL3 laboratory, and as we produced ourselves our own colony of HLA class I transgenic mice, we were limited in the number of mice required to perform these protection experiments. However, despite these conditions, we used a minimum of 5 to 7 mice to test the immunogenicity as well as the immune protection induced by the different constructs, which is the number authorized by our ethical committee of animal experimentation.

1. Table 1: The total number of SFC indicates the total number of spots per million cells for all the human donors infected with ZIKV and responding to at least 1 peptide. The Average SFC corresponds to the average number of spots per million cells in the responding donors against this peptide. The T cell response against ZIKV peptides as well the control background response (not shown in the Table 1) for each donor was performed on thawed PBMCs after *in vitro* stimulation with ZIKV overlapping peptides or with medium alone (Ref. 22). Values were calculated by subtracting the number of spots detected in the control wells stimulated with medium alone. Values were considered positive if they were equal or greater than 20 spots and at least three times above the means of the control wells with medium alone. A few sentences have been added to the methods in the ELISpot assay section.

For the peptide NS1.2 989-1003, only 2 donors (10% of the donors tested) responded to this peptide, with 295 and 320 SFC/million cells. The average response is 307. This peptide sequence was selected in the ZIKV-NS poly-epitope, but we did not observe any T cell response against this peptide in HLA-A*2402 nor in HLA-B*0702 transgenic mice.

2. For the T cell response to ConA, panels A, B and C were performed in the same ELISpot assay, explaining why the response to the medium alone or to the ConA was shown only in panel C. Different ELISpot assays were performed in panels D, E and F, with the negative (medium alone) and positive controls (ConA) shown in each of these panels. We have added this clarification in Fig. 1 legend, as well as the x-axis legend in the figure legend.

Fig. 1 represents the T cell response to overlapping peptides covering the whole ZIKV-NS or DENV1-NS poly-epitopes. In the HLA-A*2402 and HLA-B*0702 transgenic mice, we have detected no T cell response to peptides derived from the Capsid, or the NS1 of ZIKV-NS. Knowing that C and NS1 contain several epitopes detected in the human, this suggests that these C or NS1 epitopes are restricted by other HLA class I alleles or that they are restricted by HLA class II alleles.

**Minor modifications:**

**Reviewer #1:**

1. Aedes line 25 page 2 and line 7 page 3 have been italicized in the revised manuscript.

2. Line 23 page 3: the spelling of Envelope has been corrected the revised manuscript

3. Page 4 line 4, ADE has been defined as Antibody Dependent Enhancement

4. Fig. 5 legend, we have specified that the y axis is in Log scale.

**Summary and General comment:**

**Reviewer #1:**

Why add Capsid and NS1. As shown in Table 1, we have detected a significant number of human donors responding to antigenic peptides from the C and NS1 regions. As the restricting alleles for these peptides is unknown, we wanted to introduce these antigenic regions in the ZIKV poly-epitopes. Results show the absence of T cell response in the context of HLA-A*2402 and -B*0702 alleles (in transgenic mice). This suggests that these peptides are restricted by other HLA class I alleles than A24 or B7, or that these peptides are restricted by HLA class II molecules.

**Reviewer #2:**

Concerning **the immunogenicity**, we have also measured the phenotype of activated CD3+ CD8+ T cells, at day 10 and day 24 after the last immunization, in 2 different HLA class I transgenic mice, the HLA-A*2402 and HLA-A¨0201.

These data have been added in the revised version of the paper (Fig 4 and Supplementary Fig. S1, page 14 line 8-25).

**Efficacy**: We wanted to verify that the 704-DNA platform can lead to equivalent immunization in HLA-B*0702 transgenic mice and in HLA-B*0702 transgenic IFNAR-/- mice. Results show no significant difference in the antigenic peptides nor in the intensity of the response between IFNAR wt and IFNAR-/- mice. For challenge experiments, we used mice treated with anti-IFNAR Abs, which induces a transient blockade in IFN-a/b signaling and allows viral replication and differentiation of T cells into effector memory cells.

Regarding the question of quantification of the viral load, as described in the Methods section, the viral RNA is quantified, after extraction from the serum of infected mice, and cDNA copies of viral RNA are synthesized using the MMLV cDNA synthesis kit. cDNA is then quantified using specific probes and primers for ZIKV and DENV2. We have modified the y axis of Fig. 4 legend.

We did not quantify the viral load in other organs, such as brain, testes, liver or spleen, which represents a limitation of this study.

The number of mice used for these protection studies were 5 to 6 animal per type of immunization and challenge. Each panel is representative of 3 independent experiments, in which for each experiment, a total of 5 and 4 HLA-A*2402 mice received the ZIKV-NS and the empty vector, respectively (panel A), 6 and 6 HLA-B*0702 mice received the ZIKV-NS and the empty vector, respectively, 5 and 6 HLA-B*0702 mice received the DENV1-NS and empty vector, respectively, and 6 and 6 HLA-B*0702 mice received the ZIKV-NS construct, respectively.

We look forward to your consideration of the revised manuscript and hope that you and your reviewers share our enthusiasm for our results.

Sincerely,

Dr Claude Roth

Department of Global Health

Institut Pasteur

Mail: claude.roth@pasteur.fr
