# Supplementary material for: Zika virus T-cell based 704/DNA vaccine promotes protection from Zika virus infection in the absence of neutralizing antibodies
Source: PLoS Negl Trop Dis. 2024 Oct 17;18(10):e0012601. doi: 10.1371/journal.pntd.0012601 (PMC11521268; doi:10.1371/journal.pntd.0012601)
Supplement: Attachment — Submitted filename: Responses to the reviewers.docx [file pntd.0012601.s006.docx]

**Plos Negleted Tropical Diseases**	Paris September 13, 2024

Dear Editor,

Thank you for the opportunity to revise and to resubmit our manuscript "Zika virus T-cell based 704/DNA vaccine promotes protection from Zika virus infection in the absence of neutralizing antibodies" (PNTD-D-24-00451). We have modified the manuscript according to the review recommendations. I also changed my email address, for [clroth75@gmail.com](mailto:clroth75@gmail.com), as the address at Pasteur ([claude.roth@pasteur.fr](mailto:claude.roth@pasteur.fr) will not be available from January 1^st^, 2025.

Below we have detailed how we addressed the reviewer’s concerns.

**Minor issues raised by Reviewer #1:**

Page 3

- Line 7: The sentence “ZIKV is transmitted …” has been changed to “ZIKV transmission occurs via the bite of an infected *Aedes* mosquito and in some cases through human sexual contact or the maternal fetal route”.

- Line 19: More specifically has been removed.

- Line 21: the sentence “specially between ZIKV and DENV4, they share 67-68% sequence identity in the NS3 and NS5 proteins” has been changed to “specially between ZIKV and DENV4, sequence identity is 67-68% for the NS3 and NS5 proteins”.

- Line 35-37: The sentence has been slightly modified to “The risk to develop a symptomatic and severe DENV infection, especially DENV2, depends on the quality of neutralizing antibodies and the level of pre-existing anti-DENV or anti-ZIKV antibodies, with a higher risk associated with lower levels of pre-existing antibodies to DENV or ZIKV”.

Page 4

- Line 21: “protecting” has been changed to “protective”.

- Line 28: “CD4 T cell” has been changed to “CD4 T cells”.

Page 5

- Line 14: “The ZIKV proteome” has been added

- Line 23: “In has been removed”.

**Other minor issues:**

- In the results section, page 10 line 12: the number of peptides has been changed to 19

- Figures 5 and 6 have been placed in the correct order

- Page 14, line 8-9: the DENV1-NS poly-epitope has been added in the title.

We look forward to your consideration of the revised manuscript and hope that you and your reviewers share our enthusiasm for our results.

Sincerely,

Dr Claude Roth

Department of Global Health

Institut Pasteur

Mail: [clroth75@gmail.com](mailto:clroth75@gmail.com)
